# Central Administration of 1-Deoxynojirimycin Attenuates Hypothalamic Endoplasmic Reticulum Stress and Regulates Food Intake and Body Weight in Mice with High-Fat Diet-Induced Obesity

**DOI:** 10.1155/2017/3607089

**Published:** 2017-07-17

**Authors:** Jongwan Kim, Eun-Young Yun, Fu-Shi Quan, Seung-Won Park, Tae-Won Goo

**Affiliations:** ^1^Department of Anatomy, Graduate School of Dongguk University College of Medicine, 123 Dongdae-ro, Gyeongju-si 38066, Republic of Korea; ^2^Graduate School of Integrated Bioindustry, Sejong University, 209 Neungdong-ro, Gwangjin-gu, Seoul 05006, Republic of Korea; ^3^Department of Medical Zoology, Kyung Hee University, Seoul 02447, Republic of Korea; ^4^Department of Biotechnology, Catholic University of Daegu, Gyeongsan-si, Gyeongsangbuk-do 38430, Republic of Korea; ^5^Department of Biochemistry, Dongguk University College of Medicine, 123 Dongdae-ro, Gyeongju-si 38066, Republic of Korea

## Abstract

The *α*-glucosidase inhibitor, 1-deoxynojirimycin (DNJ), is widely used for its antiobesity and antidiabetic effects. Researchers have demonstrated that DNJ regulates body weight by increasing adiponectin levels, which affects energy intake and prevents diet-induced obesity. However, the mechanism by which centrally administered DNJ exerts anorexigenic effects has not been studied until now. We investigated the effect of DNJ in the hypothalamus of mice with high-fat diet-induced obesity. Results showed that intracerebroventricular (ICV) administration of DNJ reduced hypothalamic ER stress, which activated the leptin-induced Janus-activated kinase 2 (JAK2)/signal transducers and activators of transcription 3 (STAT3) signaling pathway to cause appetite suppression. We conclude that DNJ may reduce obesity by moderating feeding behavior and ER stress in the hypothalamic portion of the central nervous system (CNS).

## 1. Introduction

Obesity is increasingly becoming a public health problem worldwide [1]. It is caused by an energy imbalance characterized by high energy intake relative to energy expenditure, as well as established risk factors such as type 2 diabetes, cardiovascular disease, atherosclerosis, stroke, and dyslipidemia [2]. In the central nervous system (CNS), the hypothalamus plays a critical role as the primary regulator of feeding behavior and energy homeostasis by regulating appetite-related neuropeptides and neuronal excitations [3, 4]. Dysfunction of the hypothalamus leads to energy imbalance, which has been recognized as a possible pathogenic mechanism of obesity [5, 6].

Research suggests that hypothalamic endoplasmic reticulum (ER) stress causes hypothalamic dysfunction and neuronal apoptosis, which leads to feeding behavior disorders associated with obesity and diabetes [7–9]. Indeed, hypothalamic ER stress and leptin resistance are associated with increased energy intake and body weight [10, 11]. The two factors are closely linked: Hypothalamic ER stress has been shown to play a causal role in the development of leptin resistance and obesity [10, 12]. Moreover, in vitro and in vivo treatment with ER stress inducers have been shown to promote hypothalamic ER stress and attenuate leptin-induced phosphorylation of signal transducer and activator of transcription 3 (STAT3) proteins, important because phosphorylation of STAT3 at Try1138 plays a key role in mediating the effects of leptin on energy balance [12–15].

Leptin, a substance primarily synthesized and secreted by white adipose tissue, is important for regulating food intake and energy expenditure in the CNS [16–18]. Leptin receptors (LepRs) are densely packed in the hypothalamus [19, 20]. The binding of leptin to LepRs leads to phosphorylation of Janus-activated kinase 2 (JAK2), which in turn phosphorylates several tyrosine residues of LepR, thus activating different signaling pathways and physiological functions [14, 19–21].

In the CNS, the arcuate nucleus (ARC) of the hypothalamus is crucial for regulating feeding and energy homeostasis. The ARC contains two major subpopulations of neurons for controlling appetite: orexigenic neurons that coexpress neuropeptide Y (NPY) and agouti-related protein (AgRP) and anorexigenic neurons that express peptide proopiomelanocortin (POMC) [22, 23]. Within the ARC, leptin directly regulates energy intake by inhibiting NPY/AgRP neurons and stimulating POMC neurons [23–26]. In addition, hypothalamic adenosine monophosphate- (AMP-) activated protein kinase (AMPK) maintains energy homeostasis by regulating appetite and nutrient metabolism through intracellular signaling [27]. This kinase also regulates energy homeostasis through orexigenic signals (fasting, ghrelin, etc.) and anorexigenic signals (leptin, insulin, feeding, etc.) in the hypothalamus [27–30].

The *α*-glucosidase inhibitor, 1-deoxynojirimycin (DNJ), has been isolated from the leaves and roots of the mulberry tree* (Morus alba)*, as well as silkworm* (Bombyx mori)* larvae and several microorganisms including* Bacillus subtilis *and* Streptomyces *[31–34]. 1-Deoxynojirimycin is being investigated for potential antidiabetic and antiobesity effects because it reduces body weight and serum hyperglycemia and enhances carbohydrate metabolism and insulin tolerance. All of these effects improve diabetic conditions by inhibiting *α*-glucosidase activity and preventing absorption of glucose in the small intestinal brush border [31, 32, 35]. Researchers have recently reported that DNJ has significant antiobesity effects in Otsuka Long Evans Tokushima Fatty (OLETF) rats and diet-induced obese mice [31]. 1-Deoxynojirimycin also reportedly stimulates the production of adiponectin in cultured 3T3-L1 adipocytes and increases expression of adiponectin mRNA in adipose tissues [32, 36].

1-Deoxynojirimycin (DNJ) and N-alkylated DNJ [N-butyl DNJ (NB-DNJ) and N-nonyl DNJ (NN-DNJ)] were the major iminosugars [37]. The hydrophilic DNJ is a potent *α*-glucosidase inhibitor in comparison to the more hydrophobic NB-DNJ and NN-DNJ compounds [38, 39], and DNJ was far less toxic than the lipophilic NB-DNJ [40, 41]. The above contents are explained in the manuscript.* N*-Butyldeoxynojirimycin (NB-DNJ), an inhibitor of *α*-glucosidases, reduces body weight and food intake via central appetite suppression [42]. Orally administered NB-DNJ has been shown to cross the blood brain barrier [43].

However, the mechanisms by which centrally administered DNJ affects leptin and ER stress have yet to be studied. Therefore, we investigated whether centrally administering DNJ into the hypothalamus of high-fat diet- (HFD-) fed obese mice would reduce ER stress and lead to anorexigenic effects that attenuate diet-induced obesity (DIO).

## 2. Methods

### 2.1. Reagents and Cells

1-Deoxynojirimycin was purchased from Sigma-Aldrich (St Louis, MO, USA). QGreenTM 2x SybrGreen qPCR Master Mix was obtained from CellSafe (Suwon, Korea). Mouse hypothalamic GT1-7 cells were grown in Dulbecco's modified Eagle's media (Gibco, Rockville, MD, USA) and added with 10% heat-inactivated fetal bovine serum (FBS; Gibco), 100 U/mL penicillin, and 100 *μ*g/mL streptomycin (Gibco).

### 2.2. MTT Assay

We followed the methods described by Kim et al. (2016) to measure cell viability [44]. Cell viability was measured by 3-(4,5-dimethylthiazol-2-yl)-2,5-diphenyltetrazolium bromide (MTT) assay. Hypothalamic neuronal GT1-7 cells were seeded at a density of 1 × 10^4^ cells per well in a 96-well plate in triplicate. Following treatment, culture media were got rid of and MTT (0.5 mg/mL) was added, after which samples were incubated at 37°C for 2 h in a CO_2_ incubator. Absorbance was measured at 570 nm using a microplate reader (Anthos Labtec Instruments, Wals, Austria) after dissolving the insoluble crystals in dimethyl sulfoxide (DMSO).

### 2.3. Animals

Male C57BL/6J mice (7 weeks of age) were purchased from Japan SLC (Hamamatsu, Japan). The mice were permitted free access to standard chow diet and water ad libitum for 12 weeks. To induce DIO, 8-week-old mice were fed with a HFD (60% fat; D12492; Research Diets, New Brunswick, NJ, USA) for 12 weeks [44]. Lean control mice were fed with a low-fat diet (LFD; 10% fat; D12450B; Research Diets) for the same period. All mice were maintained in a room at controlled temperature (23  ±  1°C), under a 12-hour light/12-hour dark cycle with free access to water and food. All procedures were approved by the Institution Animal Care and Use Committee of the College of Medicine, Dongguk University, and followed the Principles of Laboratory Animal Care (NIH, Washington, DC, USA) [44].

### 2.4. Cannula Implantation and DNJ Administration

We followed the method outlined by Kim et al. (2016) [44]. A 26-gauge stainless steel guide cannula (5 mm below the pedestal; C315G; Plastics One, Roanoke, VA, USA) implanted into the third ventricle of the mice under stereotaxic control using a stereotaxic apparatus (coordinates from Bregma: anteroventral, −1.8 mm; lateral, 0.0 mm; dorsoventral, 5.0 mm) through a hole created in the skull with a micro driller. The cannula was secured to the skull with dental cement and capped with a dummy cannula (C315DC; Plastic One) that extended 0.5 mm below the guide cannula. The animals were weighed daily, and any animal showing signs of illness or weight loss was removed from the study and euthanized. At 7 days after intracerebroventricular (ICV) cannulation, the HFD (*n* = 14) and LFD (*n* = 14) mice were separated into two groups. The first group of HFD (*n* = 7) and LFD mice (*n* = 7) was immersed with 1 *μ*L of sterile distilled water as a vehicle, while the second group of HFD (*n* = 7) and LFD (*n* = 7) mice was immersed with 1 *μ*L of DNJ (50 *μ*g/mL). All ICV injections were executed using a 33-gauge internal cannula (C315I, Plastic One) that extended 0.5 mm below the guide cannula and was connected by a cannula connector to a 5 *μ*L Hamilton syringe. The solutions were immersed over 5 min. At 6 h after DNJ infusion, the hypothalamus was dissected, immediately frozen in liquid nitrogen, and stored at −80°C until further processing.

### 2.5. Timeline of the Experimental Procedure

**Figure 1:**
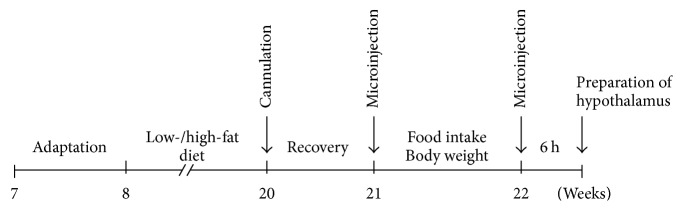
Timeline of the experimental procedure for measuring the effect of centrally administered 1-deoxynojirimycin (DNJ) on food intake and body weight in high-fat diet- (HFD-) fed mice.

Seven-week-old male C57BL/6J mice were purchased from Japan SLC (Hamamatsu, Japan). For adaptation, the mice were allowed chow diet and water ad libitum for a week. At 8 weeks of age, the study mice were fed with a HFD for 12 weeks to create DIO. The control mice were fed with a LFD for 12 weeks. Subsequently, the LFD- and HFD-fed mice were implanted with a stainless steel cannula into the third ventricle using a stereotaxic apparatus according to the manufacturer's instructions. The mice were allowed to recover for a week. At 21 weeks of age, food intake and body weights were analyzed after ICV injections of either DNJ or the vehicle. Six hours after injection, the mice's hypothalami were dissected and flash-frozen in liquid nitrogen (Figure 1).

### 2.6. Western Blot Analysis

For detection of a specific target protein, we followed the methods of western blot analysis as described in detail in [44]. Tissues or cells were lysed in radioimmunoprecipitation assay (RIPA) lysis buffer (50 mM Tris-HCl, pH 8.0, 150 mM NaCl, 0.02% sodium azide, 0.1% SDS, 1% NP-40, 0.5% sodium deoxycholate, and 1 mM phenylmethylsulfonyl fluoride). Protein concentrations of cell lysates were determined using a BioRad protein assay kit (BioRad, Hercules, CA, USA). Equal amounts of protein were subjected to 8 to 15% sodium dodecyl sulfate polyacrylamide gel electrophoresis (SDS-PAGE) and transferred to polyvinylidene difluoride (PVDF) membranes (BioRad). The membranes were blocked with 5% skim milk and sequentially reacted with primary antibodies (mouse monoclonal anti-C/EBP-homologous protein [CHOP] antibody: 1 : 1000, Cell Signaling Technology, Danvers, MA; rabbit polyclonal anti-phospho-eukaryotic initiation factor 2 [anti-phospho-eIF2*α*] antibody: 1 : 1000, Cell Signaling Technology; rabbit polyclonal anti-eIF2*α* antibody: 1 : 1000, Cell Signaling Technology; rabbit polyclonal anti-GRP78 antibody: 1 : 1000, Cell Signaling Technology; mouse monoclonal anti-Ero1L antibody: 1 : 1000, Abnova, Taipei, Taiwan; mouse monoclonal anti-PDI antibody: 1 : 1000, Enzo Life Sciences, Inc., Plymouth Meeting, PA; rabbit polyclonal anti-cleaved poly-[adenosine diphosphate-ribose] polymerase [PARP]: 1 : 1,000; Cell Signaling Technology; rabbit polyclonal anti-cleaved caspase-3: 1 : 1000, Cell Signaling Technology; rabbit polyclonal anti-cleaved caspase-3 : 1 : 1000, Cell Signaling Technology; rabbit monoclonal anti-phospho-JAK2 [Tyr1008]: 1 : 1,000, Cell Signaling Technology; rabbit monoclonal anti-JAK2: 1 : 1000, Cell Signaling Technology; rabbit monoclonal anti-phospho-STAT3 [Tyr705]: 1 : 1000: Cell Signaling Technology; mouse monoclonal anti-STAT3: 1 : 1000; Cell Signaling Technology; rabbit polyclonal anti-phospho-AMPK*α* antibody: 1 : 1000, Cell Signaling Technology; rabbit polyclonal anti-AMPK*α* antibody: 1 : 1000, Cell Signaling Technology; *α*-tubulin antibody: 1 : 2000, Sigma-Aldrich; and horseradish peroxidase- [HRP-] conjugated secondary antibody: 1 : 1000; anti-mouse and rabbit-IgG antibodies: Amersham Biosciences) followed by detection using an enhanced chemiluminescence (ECL) detection kit (Invitrogen, Waltham, MA).

### 2.7. Reverse Transcription-PCR

We followed the method outlined by Kim et al. (2016) [44]. Total RNA was isolated from tissues using an RNeasy Mini Kit (Qiagen, Hilden, Germany) according to the manufacturer's instructions. An aliquot of RNA was separated by 1% agarose gel electrophoresis to confirm integrity of the RNA sample. cDNA was synthetized using Moloney murine leukemia virus (M-MLV) reverse transcriptase (Promega, Madison, WI) and oligo (dT) primers after DNase I treatment (Invitrogen, Life Technologies). Real-time polymerase chain reaction (PCR) was executed using the specific primer set in Table 1. Traditional PCR amplification was performed at an annealing temperature of 60°C for 27 cycles using the specific primer set in Table 1. For analysis of PCR products, 10 *μ*L of each PCR product was separated by a 1–2.5% agarose gel and detected under UV light. Glyceraldehyde 3-phosphate dehydrogenase (GADPH) was used as an internal control.

### 2.8. Data Analysis

All data are shown as the mean ± standard deviation (SD). Comparisons between two groups were performed using Student's* t*-test. Comparisons between three or more groups were carried out using one-way analysis of variance (ANOVA) followed by Dunnett's test. SPSS version 18.0 K (SPSS Inc., Chicago, IL, USA) was used for analyses, and *P* value differences of <0.05 were considered statistically significant.

## 3. Results

### 3.1. 1-Deoxynojirimycin Reduces ER Stress and Activates Leptin-Induced Signaling in Mouse Hypothalamic Neuronal GT1-7 Cells

**Figure 2:**
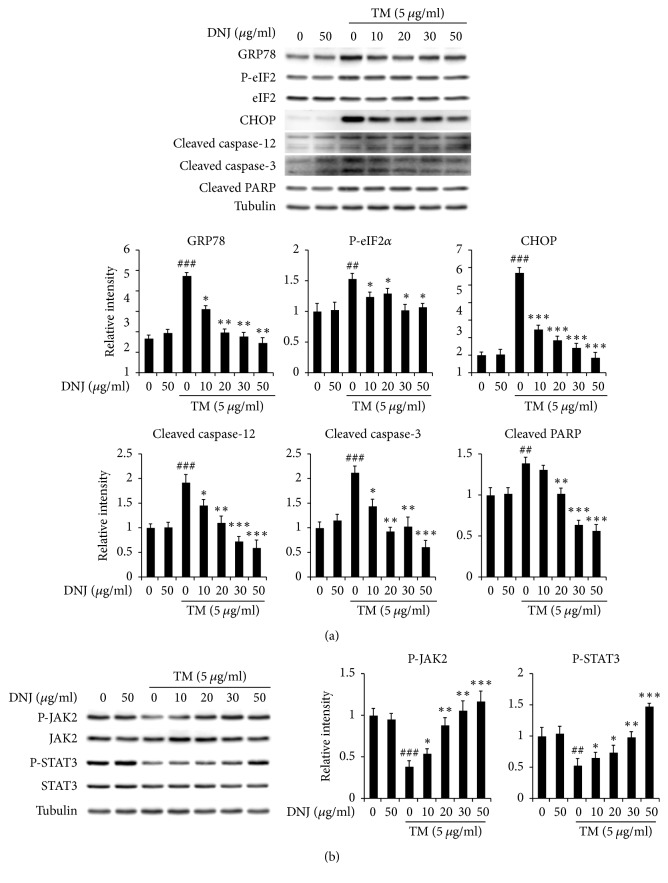
Effect of 1-deoxynojirimycin (DNJ) on endoplasmic reticulum (ER) stress and leptin signaling in mouse hypothalamic neuronal GT1-7 cells. (a) GT1-7 cells were treated with tunicamycin (TM; 5 *μ*g/mL) as well as DNJ (10–50 *μ*g/mL) for 6 h, after which ER stress and apoptosis responsive markers were measured. (b) GT1-7 cells were treated with TM (5 *μ*g/mL) as well as DNJ (10–50 *μ*g/mL) for 6 h, after which leptin signaling markers were measured. The results of densitometric analysis are the means ± SDs (*n* = 3). ^#^*P* values (^##^*P* < 0.01; ^###^*P* < 0.001) indicate statistical significance compared to that of vehicle (1 *μ*L of distilled water; DW). ^*∗*^*P* values (^*∗*^*P* < 0.05; ^*∗∗*^*P* < 0.01; ^*∗∗∗*^*P* < 0.001) indicate statistical significance compared to that of TM (5 *μ*g/mL).

We tested the cytotoxicity of DNJ on hypothalamic neuronal GT1-7 cells by treating the cells with various concentrations of DNJ (10–1,000 *μ*g/mL) and then analyzing them using an MTT assay. 1-Deoxynojirimycin showed no cytotoxic effects on GT1-7 cells (Figure S1 in Supplementary Material available online at https://doi.org/10.1155/2017/3607089). Next, we conducted western blot analysis on GT1-7 cells that were treated with various concentrations of DNJ (10–50 *μ*g/mL) and the N-glycosylation inhibitor tunicamycin (TM; 5 *μ*g/mL) to investigate whether DNJ could regulate hypothalamic ER stress and leptin signaling pathways. The results showed that DNJ decreased expression of ER stress markers (e.g., 78 kDa glucose-regulated protein, GRP78; CHOP; and phospho-eIF2) and apoptosis responsive markers (e.g., caspase-3 and caspase-12) in a dose-dependent manner. In contrast, treating GT1-7 cells with DNJ caused a dose-dependent increase in leptin-induced signaling by proteins such as phospho-JAK2 and phospho-STAT3 in comparison to treatment with TM alone. Based on these results, we concluded that DNJ significantly induces expression of the leptin signaling pathway by reducing ER stress (Figures 2(a) and 2(b)). Therefore, we proceeded to investigate whether central administration of DNJ affects hypothalamic ER stress, appetite-related neuropeptides, and leptin signaling in HFD-fed obese mice.

### 3.2. Hypothalamic Endoplasmic Reticulum Stress Dramatically Increased in HFD-Fed Obese Mice

**Figure 3:**
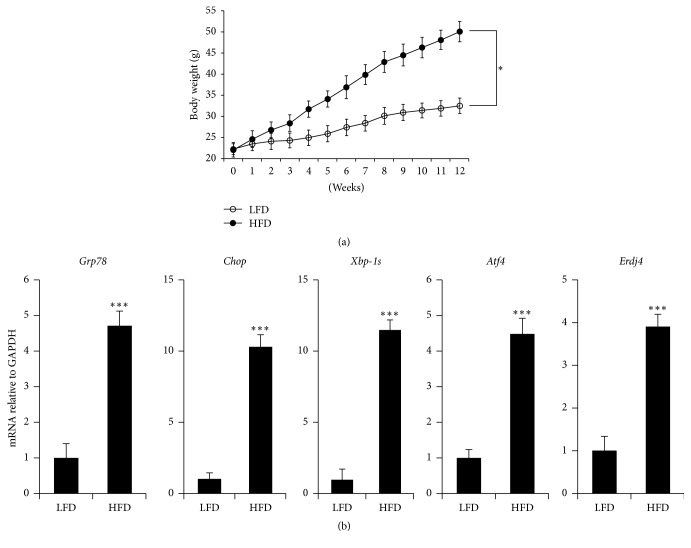
Increased hypothalamic endoplasmic reticulum (ER) stress in high-fat diet- (HFD-) fed mice. (a) Time dependence of body weight in low-fat diet- (LFD-) and HFD-fed mice. At 12 weeks of age, HFD-fed mice showed significantly higher body weight than those fed with LFD. (b) mRNA expression levels of ER stress response markers in LFD- and HFD-fed mice. HFD dramatically upregulated the mRNA expression levels of ER stress response markers in obese mice. The results are the means ± SDs (*n* = 7). *P* values (^*∗*^*P* < 0.05; ^*∗∗∗*^*P* < 0.001) indicate significant differences compared to those of LFD-fed mice.

To investigate whether obesity induces hypothalamic ER stress in mice, we fed male C57BL/6J mice with a HFD (60% kcal from fat) and LFD (10% kcal from fat) for 12 weeks. The body weight gained by HFD-fed mice was significantly higher than that gained by LFD-fed mice (Figure 3(a)). We isolated mRNA from the hypothalamus of the LFD- and HFD-fed mice and performed quantitative PCR analysis to analyze for ER stress responsive markers such as* Grp78*,* Chop*, spliced X-box binding protein 1* (Xbp-1s)*, activating transcription factor 4* (Atf4)*, and ER* DnaJ* homolog 4* (Erdj4)*. The expression levels of all the ER stress responsive markers that we studied dramatically increased in HFD-induced mice (Figure 3(b)). Taken together, these results indicate that HFDs induce hypothalamic ER stress, which may then activate the unfolded protein response (UPR) signaling pathway in hypothalamic cells.

### 3.3. Central Administration of DNJ Reduces Hypothalamic ER Stress

**Figure 4:**
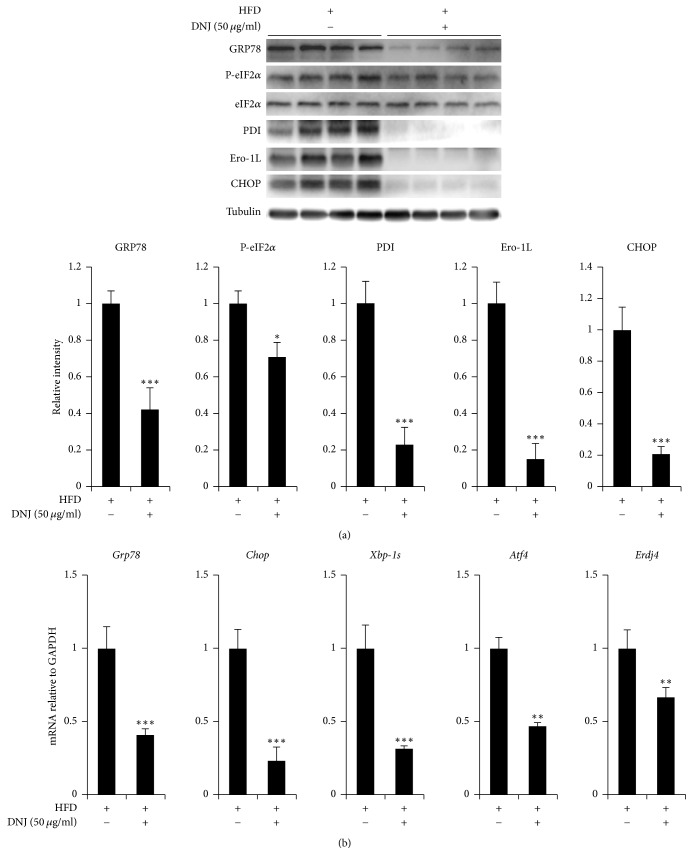
Effect of central administration of 1-deoxynojirimycin (DNJ) on endoplasmic reticulum (ER) stress response markers and ER chaperones/foldase expression in high-fat diet- (HFD-) fed mice. (a) Effects of intracerebroventricular (ICV) administration of 50 *μ*g/mL DNJ (1 *μ*L) on hypothalamic ER stress responsive markers and ER chaperones/foldases. (b) Effects of ICV administration of 50 *μ*g/mL DNJ (1 *μ*L) on hypothalamic mRNA expression of hypothalamic ER stress responsive markers. The results are the means ± SDs (*n* = 10 per group). The results of densitometric analysis are the means ± SDs (*n* = 7). *P* values (^*∗*^*P* < 0.05; ^*∗∗*^*P* < 0.01; ^*∗∗∗*^*P* < 0.001) indicate significant differences compared with vehicle (1 *μ*L of distilled water; DW). HFD/DNJ (+/−), central administration of vehicle of HFD-fed group. HFD/DNJ (+/+), central administration of DNJ of HFD-fed group.

We centrally administered 50 *μ*g/mL DNJ (1 *μ*L) or vehicle as a control into the third ventricle of HFD-fed obese mice and LFD-fed control mice. The expression levels of ER stress responsive markers (phospho-eIF2*α* and CHOP) and ER chaperones/foldases (binding immunoglobulin protein, GRP78; protein disulfide isomerase, PDI; and endoplasmic reticulum oxidoreductin 1L, Ero1L) dramatically declined in the hypothalamus of HFD-induced obese mice treated with DNJ (Figure 4(a)). Furthermore, the mRNA expression levels of ER stress responsive genes (*Grp78, chop, Xbp-1s*,* Atf4*, and* Erdj4*) were significantly lower in HFD-fed obese mice treated with DNJ than in control mice treated with the vehicle (Figure 4(b)). These results indicate that DNJ can reduce ER stress increased in the hypothalamus of obese mice fed with a HFD. However, the tendency of DNJ to affect hypothalamic ER stress did not differ in LFD-fed mice (Figure S2).

### 3.4. Central Administration of DNJ Reduces Food Intake and Body Weight Via Upregulation of Appetite-Related Anorexigenic Neuropeptide in HFD-Fed Mice

**Figure 5:**
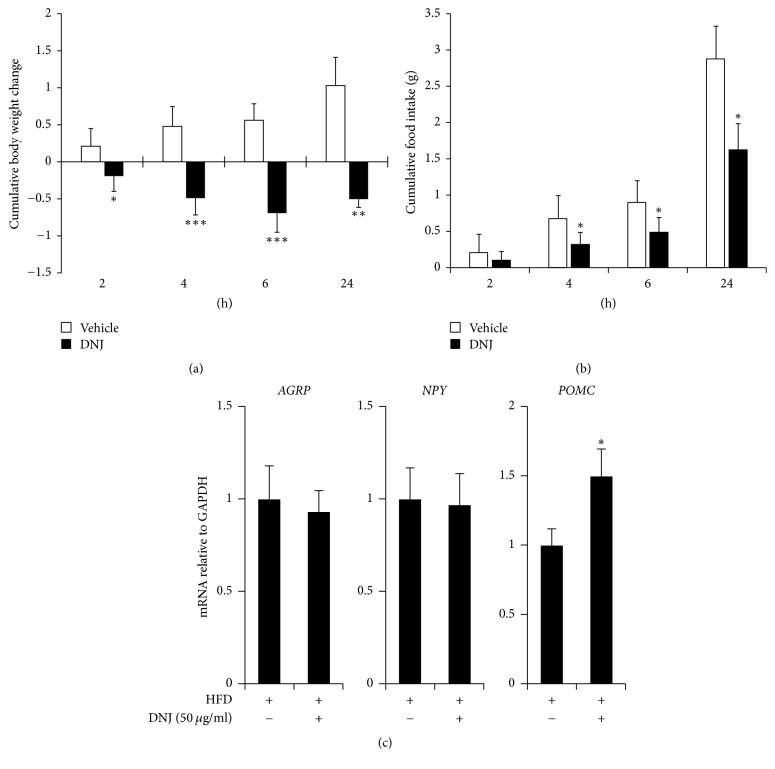
Effect of central administration of DNJ on food intake and body weight in high-fat diet- (HFD-) fed mice. (a) The average cumulative body weight change and (b) average cumulative food intake were measured in HFD-fed mice after central administration of 50 *μ*g/mL DNJ (1 *μ*L) or vehicle (1 *μ*L of distilled water; DW) during the experimental period. (c) Effects of central administration of DNJ on hypothalamic mRNA expression of neuropeptides. The results are the means ± SDs (*n* = 7 per group). ^*∗*^*P* values of < 0.05 indicate significant differences compared to those of the vehicle group (1 *μ*L of DW). HFD/DNJ (+/−), central administration of vehicle of HFD-fed group. HFD/DNJ (+/+), central administration of DNJ of HFD-fed group.

We investigated whether reduction of hypothalamic ER stress by centrally administered DNJ decreases energy intake and body weight by causing anorexigenic effects. To do this, we administered a single dose of 50 *μ*g/mL DNJ (1 *μ*L) into the third ventricle of HFD-fed obese mice (Figure 1). The results showed that central administration of DNJ consistently reduced food intake and body weight over the 24 h period after microinjection compared to that of the control group (Figures 5(a) and 5(b)). We next evaluated whether or not the anorexigenic effects of DNJ are associated with changes in ARC-derived neuropeptides. As shown in Figure 5(c), central administration of DNJ in HFD-fed mice did not cause significant changes in the mRNA expression levels of* AgRP* and* NPY*, but it did significantly increase expression of* POMC*. In contrast, DNJ had no effects on appetite-related neuropeptides in LFD-fed mice (Figure S3).

### 3.5. Central Administration of DNJ Regulates Appetite through JAK2/STAT3 Signaling Pathways

**Figure 6:**
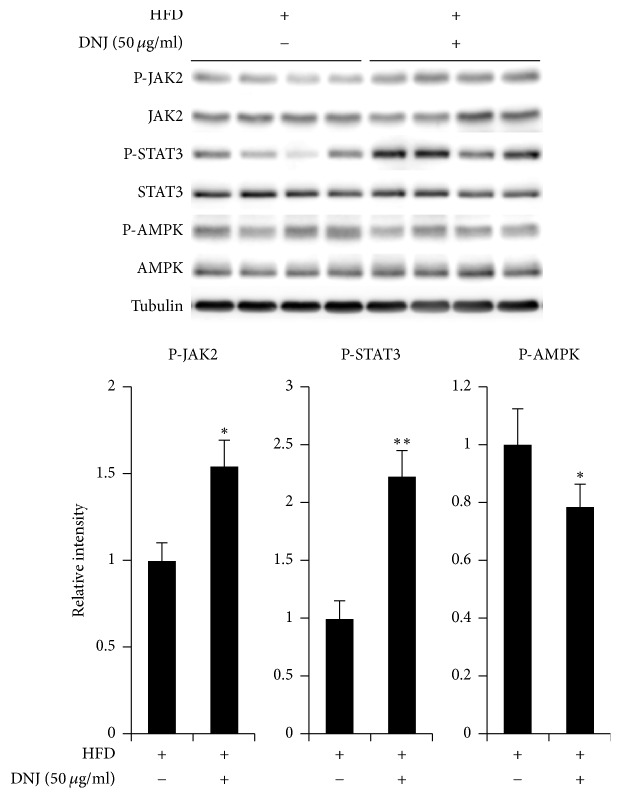
Effect of central administration of 1-deoxynojirimycin (DNJ) on leptin signaling in high-fat diet- (HFD-) fed mice. Effect of central administration of 50 *μ*g/mL DNJ (1 *μ*L) on leptin signaling pathways. The results of densitometric analysis are the means ± SDs (*n* = 7 per group). *P* values (^*∗*^*P* < 0.05; ^*∗∗*^*P* < 0.01) indicate significant differences compared with vehicle (1 *μ*L of distilled water; DW). HFD/DNJ (+/−), central administration of vehicle of HFD-fed group. HFD/DNJ (+/+), central administration of DNJ of HFD-fed group.

To determine if central administration of DNJ regulates anorexigenic effects via leptin signaling in the hypothalamus, a single dose of 5 *μ*g/mL DNJ (1 *μ*L) was administered into the third ventricle HFD-fed obese mice (Figure 6). The results indicated that central administration of DNJ significantly increased leptin-related signaling through expression of phospho-JAK2 and phospho-STAT3 in the hypothalamus of HFD-fed obese mice. In addition, central administration of DNJ reduced the hypothalamic levels of AMPK, a cellular fuel gauge, which suggests that DNJ reduces appetite through leptin signaling cascades that are triggered by reduction of ER stress in the hypothalamus of HFD-fed obese mice. However, LFD-fed mice showed no changes (Figure S4).

## 4. Discussion

Hypothalamic ER stress causes hypothalamic neuronal apoptosis and an imbalance in energy intake and expenditure that result in metabolic disorders such as obesity and diabetes. Indeed, pharmacological or genetic induction of ER stress in the hypothalamus has been shown to lead to leptin and insulin resistance, resulting in increased energy intake, hypertension, and insulin tolerance. Hypothalamic ER stress becomes elevated with obesity and diabetes. Inhibition of hypothalamic ER stress significantly attenuates these metabolic conditions [45, 46]. Therefore, treatments for attenuating hypothalamic ER stress may improve hypothalamic regulation of energy balance to control metabolic diseases.

In our study, HFDs significantly increased ER stress and the body weights of mice, and TM significantly induced ER stress and decreased the expression of leptin-induced signaling related genes (Figures 2 and 3). These results suggest that obesity may induce ER stress and leptin-related signaling in mice. Hypothalamic ER stress has been reported to play a causal role in the development of leptin resistance and obesity [10, 11, 47–49]. Activation of leptin signaling stimulates the JAK2/STAT3 pathway, which plays important roles in regulating leptin for feeding and energy expenditure [14, 19].

The antidiabetic or antiobesity effects of orally administered DNJ have been studied [31]. However, the mechanism by which DNJ directly affects the hypothalamus, an organ that plays a critical role as the primary regulator of feeding behavior and energy homeostasis by regulating appetite-related neuropeptides and neuronal excitations [31, 32, 36], has not been reported until now. We administered DNJ into the ventricle of HFD-fed obese mice to investigate the direct effect of DNJ on the hypothalamus. Central administration of DNJ significantly decreased hypothalamic ER stress, food intake, and body weight via upregulation of the anorexigenic neuropeptide* POMC*, as well as phospho-JAK2 and phospho-STAT3 (Figures 5 and 6).

In addition, centrally administered DNJ significantly decreased phospho-AMPK, a negative regulator of leptin signaling (Figure 6). In the hypothalamus, AMPK acts as a cellular fuel gauge, a central mediator that regulates feeding behavior through nutrient metabolism [50, 51]. Central administration of DNJ inhibits AMPK via the regulation of metabolic pathways [29, 50, 52]. Therefore, the inhibitory effects of hypothalamic AMPK are necessary for leptin to regulate food intake and body weight.

Accordingly, we propose that central administration of DNJ reduces food intake and body weight in HFD-fed obese mice by reducing hypothalamic ER stress, which in turn causes phosphorylation of proteins in the leptin-induced signaling pathway, which leads to upregulation of anorexigenic neuropeptides such as* POMC*. Based on these findings, we believe that DNJ is valuable for its potential therapeutic effects against various diseases caused by chronic ER stress. Because DNJ can cross the blood brain barrier [43], we recommend that it be made available for oral and intravenous administration. However, further study will be needed to clarify the effects of DNJ on regulation of energy balance during hypothalamic ER stress in obesity.

## 5. Conclusions

Our study revealed that centrally administered 1-deoxynojirimycin can reduce food intake and obesity by attenuating hypothalamic endoplasmic reticulum stress in rats. The mechanism by which this occurs involves activation of the leptin-induced signaling pathway and upregulation of anorexigenic neuropeptides. We believe that our study makes a significant contribution to the literature because although the effects of orally administered 1-deoxynojirimycin on obesity and diabetes have been studied, the mechanism by which 1-deoxynojirimycin directly acts on the hypothalamus has not been investigated until now.

## Supplementary Material

Figure S1. Cytotoxicity of DNJ in mouse hypothalamic neuronal GT1-7 cells. GT1-7 cells were treated with DNJ (1–1000 μg/mL) for 48 h, after which cell viabilities were measured with an MTT assay. The results are the means ± SDs (*n* = 3). Figure S2. Effects of central administration of 1-deoxynojirimycin (DNJ) on endoplasmic reticulum (ER) stress responsive markers and ER chaperone/foldase expression in LFD-fed mice. (A) Effects of intracerebroventricular (ICV) administration of 50 μg/mL DNJ (1 μL) on hypothalamic ER stress responsive markers and ER chaperones/foldases. (B) Effects of ICV administration of 50 μg/mL DNJ (1 μL) on hypothalamic mRNA expression of hypothalamic ER stress responsive markers. The results of densitometric analysis are the means ± SDs (*n* = 7). LFD/DNJ (+/−), central administration of vehicle of LFD-fed group. LFD/DNJ (+/+), central administration of DNJ of LFD-fed group. Figure S3. Effect of central administration of 1-deoxynojirimycin (DNJ) on food intake and body weight in low fat diet (LFD)-fed mice. (A) The average cumulative body weight change and (B) average cumulative food intake during the experimental period were measured in LFD-fed mice after central administration of 50 μg/mL DNJ (1 μL) or vehicle (1 μL of distilled water; DW). (C) Effects of central administration of DNJ on hypothalamic mRNA expression levels of neuropeptides. The results of densitometric analysis are the means ± SDs (*n* = 7). LFD/DNJ (+/−), central administration of vehicle of LFD-fed group. LFD/DNJ (+/+), central administration of DNJ of LFD-fed group. Figure S4. Effects of central administration of 1-deoxynojirimycin (DNJ) on leptin signaling in low fat diet (LFD)-fed mice. Effect of central administration of 50 μg/mL DNJ (1 μL) on leptin signaling pathways. The results of densitometric analysis are the means ± SDs (*n* = 7). LFD/DNJ (+/−), central administration of vehicle of LFD-fed group. LFD/DNJ (+/+), central administration of DNJ of LFD-fed group.

## Figures and Tables

**Table 1:** DNA primers for real-time PCR.

Mouse cDNAs	Primer sequences	GenBank accession no.
*Grp78*	Forward, 5′-GGCCTGCTCCGAGTCTGCTTC-3′	NM_022310
	Reverse, 5′-CCGTGCCCACATCCTCCTTCT-3′	

*Chop*	Forward, 5′-CCACCACACCTGAAAGCAGAA-3′	NM_007837
	Reverse, 5′-AGGTGAAAGGCAGGGACTCA-3′	

*Xbp-1s*	Forward, 5′-AGGCTTGGTGTATACATGG-3′	NM_013842
	Reverse, 5′-GGTCTGCTGAGTCCGCAGCAGG-3′	

*Atf4*	Forward, 5′-GCAAGGAGGATGCCTTTTC-3′	NM_009716
	Reverse, 5′-GTTTCCAGGTCATCCATTCG-3	

*Erdj4*	Forward, 5′-CCCCAGTGTCAAACTGTACCAG-3′	NM_013760
	Reverse, 5′-AGCGTTTCCAATTTTCCATAAATT-3′	

*AgRP*	Forward, 5′-TAGATCCACAGAACCGCGAGT-3′	NM_007427
	Reverse, 5′-GAAGCGGCAGTAGCACGTA-3′	

*NPY*	Forward, 5′-CTCCGCTCTGCGACACTAC-3′	NM_023456
	Reverse, 5′-AGGGTCTTCAAGCCTTGTTCT-3′	

*POMC*	Forward, 5′-CTGGAGACGCCCGTGTTTC-3′	NM_001278584
	Reverse, 5′-TGGACTCGGCTCTGGACTG-3′	

*GAPDH*	Forward, 5′-CTTCAACAGCAACTCCCACTCTTCC-3′	NM_001289726
	Reverse, 5′-TGGGTGGTCCAGGGTTTCTTACTCCTT-3	
