# Supplementary material for: Central Administration of 1-Deoxynojirimycin Attenuates Hypothalamic Endoplasmic Reticulum Stress and Regulates Food Intake and Body Weight in Mice with High-Fat Diet-Induced Obesity
Source: Evid Based Complement Alternat Med. 2017 Jul 17;2017:3607089. doi: 10.1155/2017/3607089 (PMC5535735; doi:10.1155/2017/3607089)
Supplement: Supplementary file 1 — Figure S1. Cytotoxicity of DNJ in mouse hypothalamic neuronal GT1-7 cells. GT1-7 cells were treated with DNJ (1–1000 μg/mL) for 48 h, after which cell viabilities were measured with an MTT assay. The results are the means ± SDs (n = 3). Figure S2. Effects of central administration of 1-deoxynojirimycin (DNJ) on endoplasmic reticulum (ER) stress responsive markers and ER chaperone/foldase expression in LFD-fed mice. (A) Effects of intracerebroventricular (ICV) administration of 50 μg/mL DNJ (1 μL) on hypothalamic ER stress responsive markers and ER chaperones/foldases. (B) Effects of ICV administration of 50 μg/mL DNJ (1 μL) on hypothalamic mRNA expression of hypothalamic ER stress responsive markers. The results of densitometric analysis are the means ± SDs (n = 7). LFD/DNJ (+/−), central administration of vehicle of LFD-fed group. LFD/DNJ (+/+), central administration of DNJ of LFD-fed group. Figure S3. Effect of central administration of 1-deoxynojirimycin (DNJ) on food intake and body weight in low fat diet (LFD)-fed mice. (A) The average cumulative body weight change and (B) average cumulative food intake during the experimental period were measured in LFD-fed mice after central administration of 50 μg/mL DNJ (1 μL) or vehicle (1 μL of distilled water; DW). (C) Effects of central administration of DNJ on hypothalamic mRNA expression levels of neuropeptides. The results of densitometric analysis are the means ± SDs (n = 7). LFD/DNJ (+/−), central administration of vehicle of LFD-fed group. LFD/DNJ (+/+), central administration of DNJ of LFD-fed group. Figure S4. Effects of central administration of 1-deoxynojirimycin (DNJ) on leptin signaling in low fat diet (LFD)-fed mice. Effect of central administration of 50 μg/mL DNJ (1 μL) on leptin signaling pathways. The results of densitometric analysis are the means ± SDs (n = 7). LFD/DNJ (+/−), central administration of vehicle of LFD-fed group. LFD/DNJ (+/+), central administration of DNJ of LFD-fed group. [file 3607089.f1.docx]

**Supporting Information**

Additional data as cited in the manuscript are presented.

**FIGURE S1.** Cytotoxicity of DNJ in mouse hypothalamic neuronal GT1-7 cells.GT1-7 cells were treated with DNJ (1–1000 µg/mL) for 48 h, after which cell viabilities were measured with an MTT assay. The results are the means ± SDs (*n* = 3).

**FIGURE S2.** Effects of central administration of 1-deoxynojiimycin **(**DNJ) on endoplasmic reticulum (ER) stress responsive markers and ER chaperone/foldase expression in LFD-fed mice. (A) Effects of intracerebroventricular (ICV) administration of 50 µg/mL DNJ (1 µL) on hypothalamic ER stress responsive markers and ER chaperones/foldases. (B) Effects of ICV administration of 50 µg/mL DNJ (1 µL) on hypothalamic mRNA expression of hypothalamic ER stress responsive markers. The results of densitometric analysis are the means ± SDs (*n* = 7). LFD/DNJ (+/-), central administration of vehicle of LFD-fed group. LFD/DNJ (+/+), central administration of DNJ of LFD-fed group.

**FIGURE S3.** Effect of central administration of 1-deoxynojiimycin **(**DNJ) on food intake and body weight in low fat diet (LFD)-fed mice. (A) The average cumulative body weight change and (B) average cumulative food intake during the experimental period were measured in LFD-fed mice after central administration of 50 µg/mL DNJ (1 µL) or vehicle (1 μL of distilled water; DW). (C) Effects of central administration of DNJ on hypothalamic mRNA expression levels of neuropeptides. The results of densitometric analysis are the means ± SDs (*n* = 7). LFD/DNJ (+/-), central administration of vehicle of LFD-fed group. LFD/DNJ (+/+), central administration of DNJ of LFD-fed group.

**FIGURE S4.** Effects of central administration of 1-deoxynojiimycin (DNJ) on leptin signaling in low fat diet (LFD)-fed mice. Effect of central administration of 50 µg/mL DNJ (1 µL) on leptin signaling pathways. The results of densitometric analysis are the means ± SDs (*n* = 7). LFD/DNJ (+/-), central administration of vehicle of LFD-fed group. LFD/DNJ (+/+), central administration of DNJ of LFD-fed group.

**FIGURE S1**

**
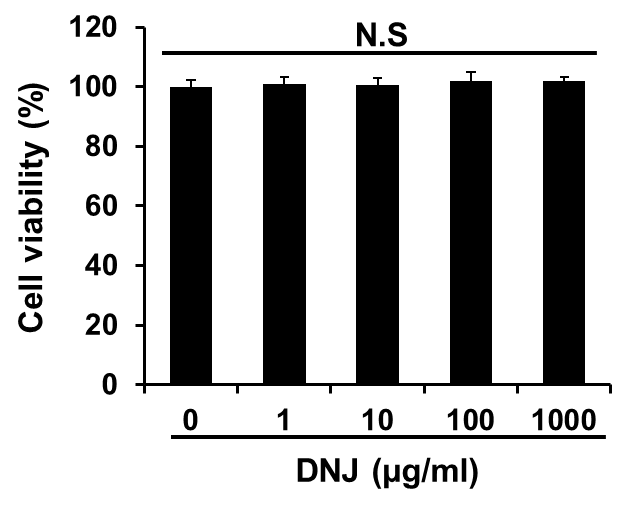
**

**FIGURE S2**

**
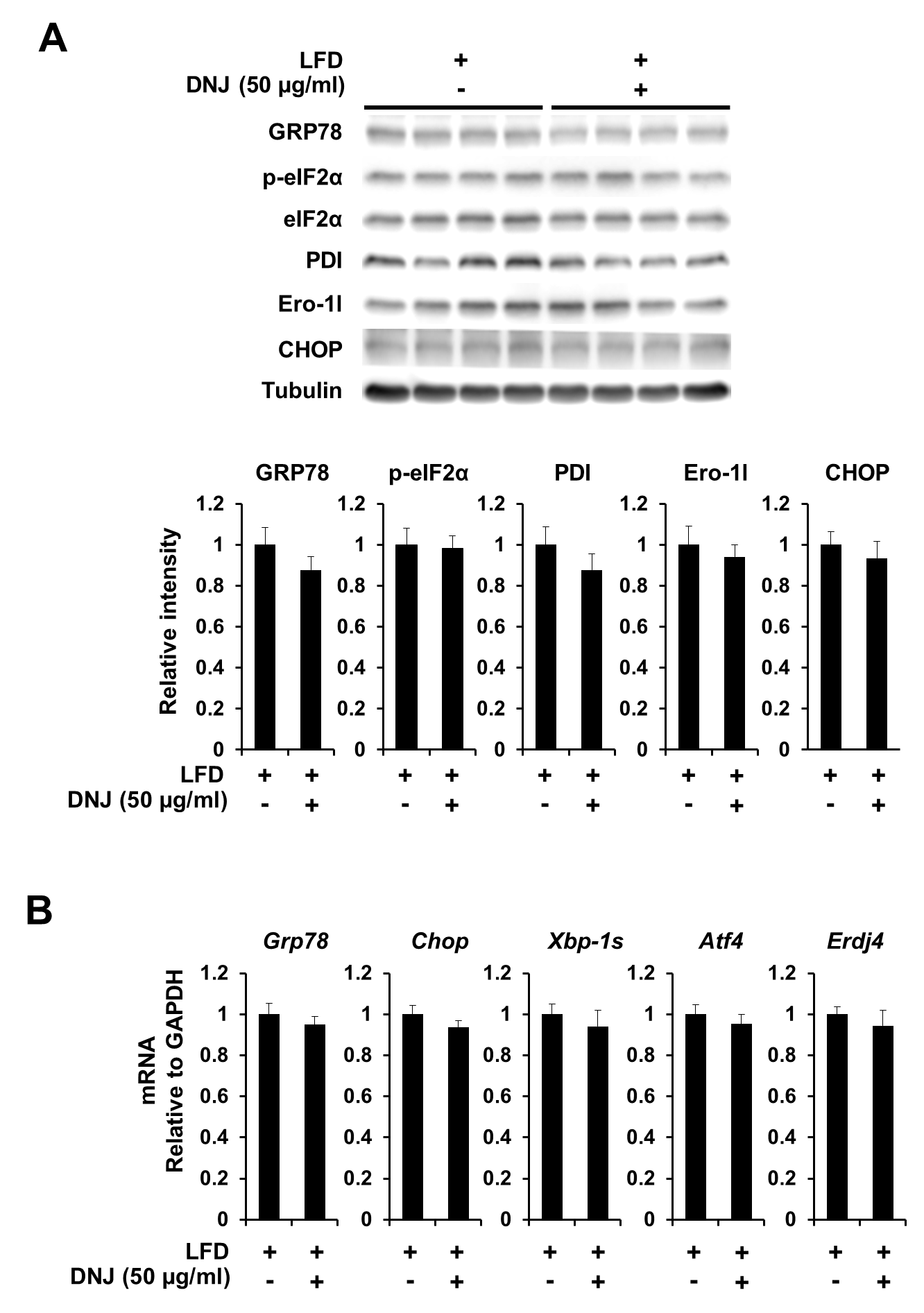
**

**FIGURE S3**

**
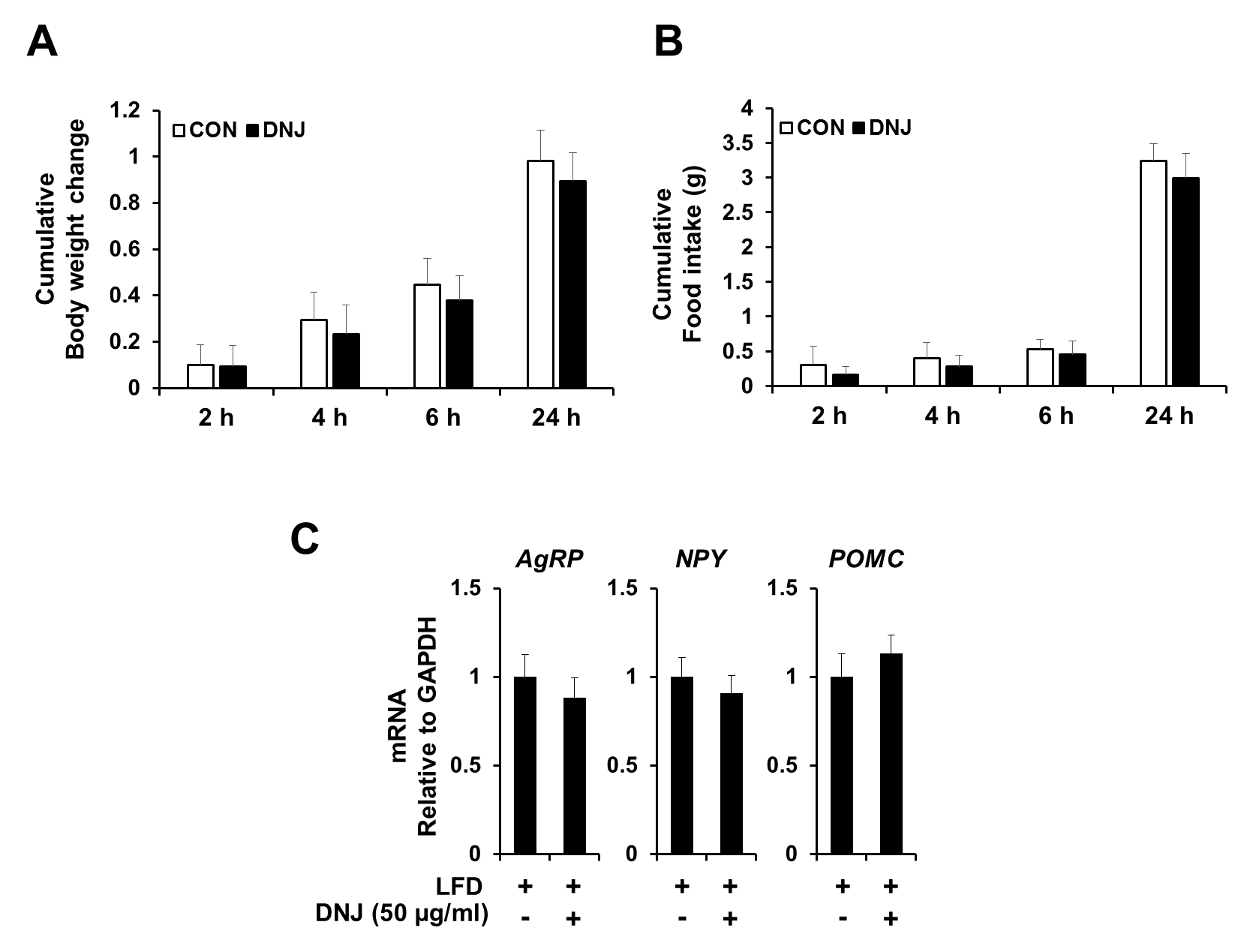
**

**FIGURE S4**

**
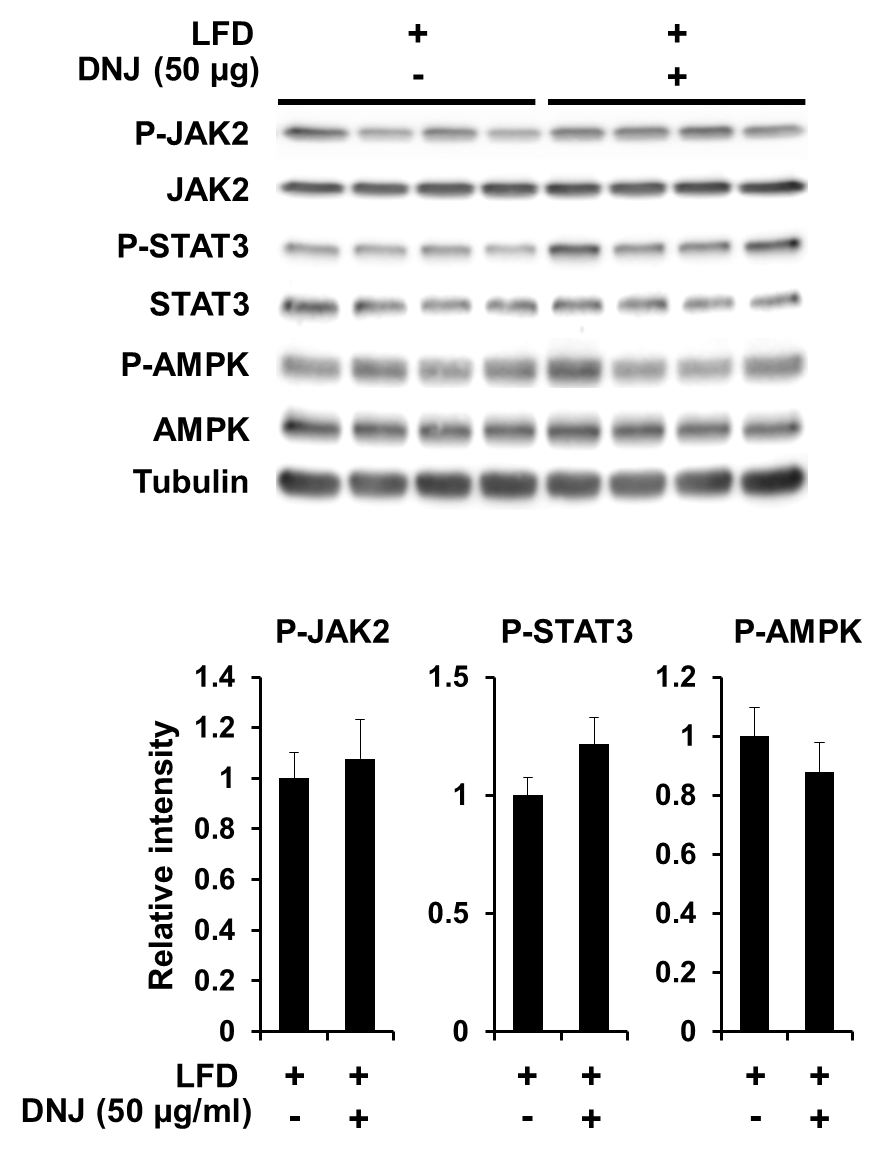
**
